# The effect of barium and strontium on activity of glucoamylase QsGH97a from *Qipengyuania seohaensis* SW-135

**DOI:** 10.1038/s41598-023-32161-y

**Published:** 2023-04-10

**Authors:** Kaijuan Wu, Xingyu Zhai, Hao Chen, Jinfeng Zheng, Zheng Yu, Xuewei Xu, Jing Huang

**Affiliations:** 1 Department of Parasitology, School of Basic Medical Science, Central South University, Changsha, 410013 Hunan China; 2 Department of Microbiology, School of Basic Medical Science, Central South University, Changsha, 410013 Hunan China; 3 Hunan Institute for Drug Control, Changsha, 410013 Hunan China; 4 Key Laboratory of Marine Ecosystem Dynamics, Ministry of Natural Resources & Second Institute of Oceanography, Ministry of Natural Resources, Hangzhou, 310012 China; 5 China-Africa Research Center of Infectious Diseases, Central South University, Changsha, 410013 Hunan China

**Keywords:** Microbiology, Ocean sciences

## Abstract

Glycoside hydrolases (GHs), the enzymes that break glycosidic bonds, are ubiquitous in the ecosystem, where they perform a range of biological functions. As an interesting glycosidase family, Glycoside hydrolase family 97 (GH97) contains α-glucosidase, α-galactosidase, and glucoamylase. Only ten members of GH97 have been characterized so far. It is critical to explore novel members to elucidate the catalytic mechanism and application potential of GH97 family. In this study, a novel glucoamylase QsGH97a from *Qipengyuania seohaensis* SW-135 was cloned and expressed in *E. coli*. Sequence analysis and NMR results show that QsGH97a is classified into GH97a, and adopts inverting mechanism. The biochemical characterization indicates that QsGH97a shows the optimal activity at 50 °C and pH 8.0. Ca^2+^ has little effect on the catalytic activity; however, the activity can be substantially increased by 8–13 folds in the presence of Ba^2+^ or Sr^2+^. Additionally, the metal content of QsGH97a assay showed a high proportion of Sr^2+^. The specific metal activity was initially revealed in glucoamylases, which is not found in other members. These results imply that QsGH97a not only is a new member of GH97, but also has potential for industrial applications. Our study reveals that Ba^2+^ or Sr^2+^ may be involved in the catalytic mechanism of glucoamylase, laying the groundwork for a more complete knowledge of GH97 and its possible industrial application.

## Introduction

Glucoamylase (GA) (EC 3.2.1.3, α-l, 4-glucan glucohydrolase), one of the most extensively utilized enzymes in industry, cleaves α-1,4-glycosidic linkages and α-1,6-glycosidic linkages from the non-reducing end of oligosaccharide substrates and starch to release β-d-glucose^1,2^. Glycoside hydrolases (GHs) are a class of enzymes that can hydrolyze glycosidic bonds in carbohydrates, and glucoamylases are mainly distributed in GH15 and GH97 family according to the carbohydrate-active enzyme (CAZy) database (http://www.cazy.org)^3^. GH family 97 (GH97), as a unique GH family, was firstly discovered in 2005^4^. In general, members of each GH family share common traits, and key residues involved in catalysis and structural stabilization are conserved within each family^5,6^. Among GH families, they cleave the glycosidic bonds based on either retaining or inverting mechanism, except in the case of GH97 enzymes They contains both inverting and retaining mechanisms^5^.

At present, GH97 consists of 3507 members, 10 of which are characterized (CAZy). Members of GH97 are confirmed to have α-glucosidase, α-galactosidase, and glucoamylase activities in CAZy database. According to multiple sequence alignment and phylogenetic analysis, GH97 can be classified into five subfamilies (97a–97e). In recent years, divergence of the molecular mechanisms and functions in GH97 has been elaborated step by step, based on the three-dimensional structure information and biological functions analysis^7–14^. Research results show that glucoamylase and α-glucosidases from GH97 contain Glu residue that acts as a catalytic base on β-strands 3 and 5 of the (β/α)8barrel following inverting mechanism, in which both HHET and HE motifs exist simultaneously, such as glucoamylase SusB in *B. thetaiotaomicron* and α-glucoside hydrolase PspAG97A in *Pseudoalteromonas* sp. K8^8,14^. On the other hand, α-galactosidases from GH97 have Asp residue as the catalytic nucleophile on β-strand 4 of the (β/α)8barrel, which involves a retaining mechanism^5^. Additionally, Ca^2+^ is required in the catalytic activity center of GH97-derived enzymes, which is not the case for some GH family-derived enzymes^15^.

It is vital to develop novel glucoamylases with excellent enzymatic properties for industrial production. GH97 members possess a completely distinct mechanism from other families; however, few enzymes in this family have been characterized. In this study, glucoamylase QsGH97a from a deep-sea bacterium *Qipengyuania seohaensis* SW-135 was heterologously expressed in *Escherichia coli* BL21 (DE3) plus strain. QsGH97a exhibited the greatest activity at 50 °C and pH 8.0. Most importantly, this study reveals that Sr^2+^ or Ba^2+^ can significantly enhance the catalytical activity and thermostability, implying that the GH97 enzymes may have additional unexplored characteristics. These results contribute to our understanding of GH97 enzymes, and provide industrial application potential for extracting glucoamylase from marine bacteria.

## Results

### Sequence analysis of QsGH97a protein

**Figure 1:**
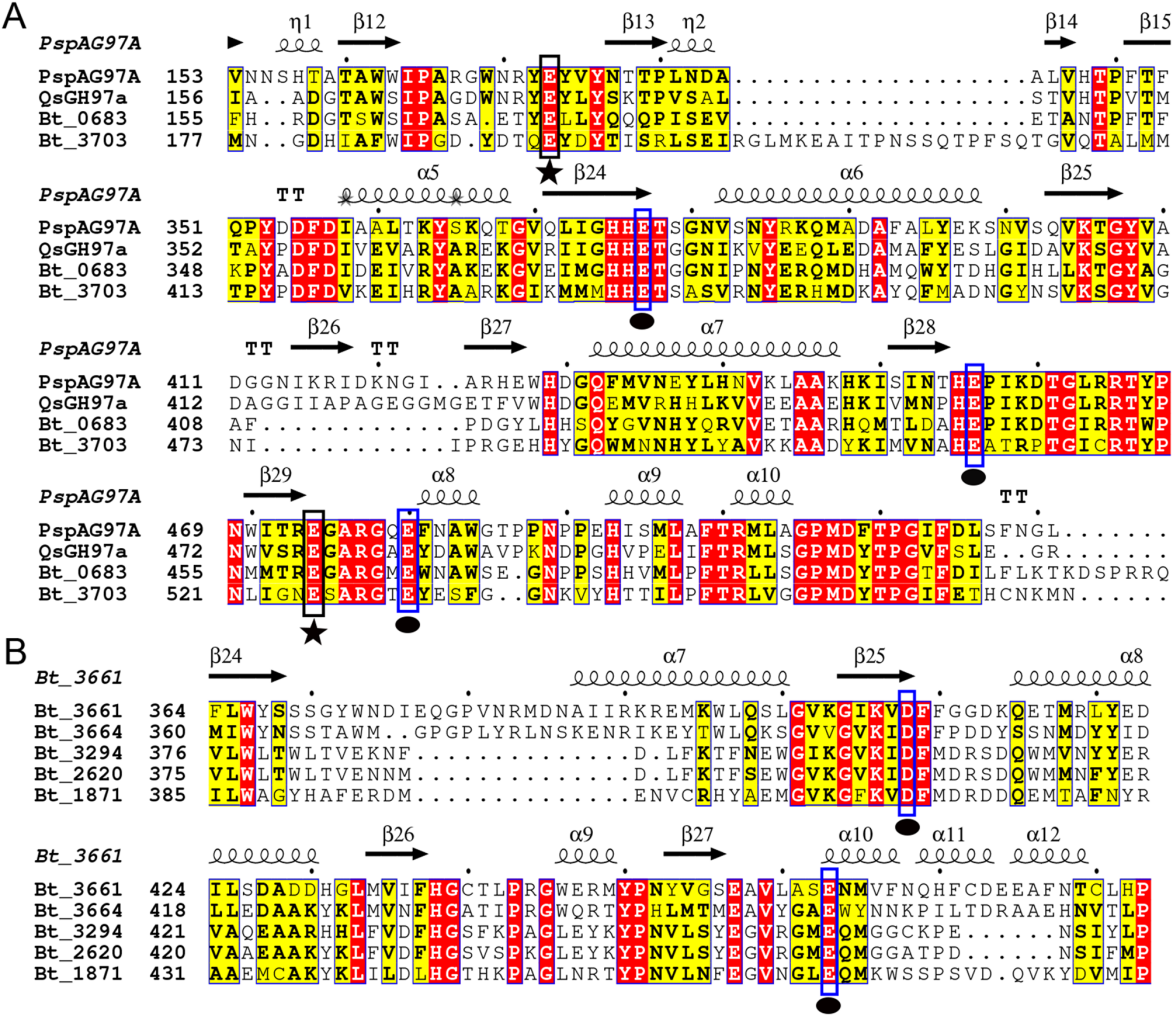
The analysis of multiple sequence alignments in GH97. (**A**) The result of acid sequence alignments in inverting enzymes of GH97. Key Glu residues marked in blue (dark circle) are the conserved catalytic sites of inverting enzymes and Glu residues shown in black (dark star) are the sites coordinating with Ca^2+^. (**B**) The result of acid sequence alignments in retaining enzymes of GH97. Important residues marked in blue (dark circle) are the conserved catalytic sites of retaining enzymes. PspAG97A**,** α-glucoside hydrolase from *Pseudoalteromonas* sp. K8; Bt_0683, α-glucosidase from *B. thetaiotaomicron* VPI-5482; Bt_3703, glucoamylase SusB from *B. thetaiotaomicron* VPI-5482; Bt_3661, bifunctional β-L-arabinopyranosidase/α-galactosidase from *B. thetaiotaomicron* VPI-5482; Bt_3664, α-galactosidase from *B. thetaiotaomicron* VPI-5482; Bt_3294, α-galactosidase from *B. thetaiotaomicron* VPI-5482; Bt_2620, α-mannan α-galactosidase from *B. thetaiotaomicron* VPI-5482; Bt_1871, α-galactosidase from *B. thetaiotaomicron* VPI-5482.

**Figure 2:**
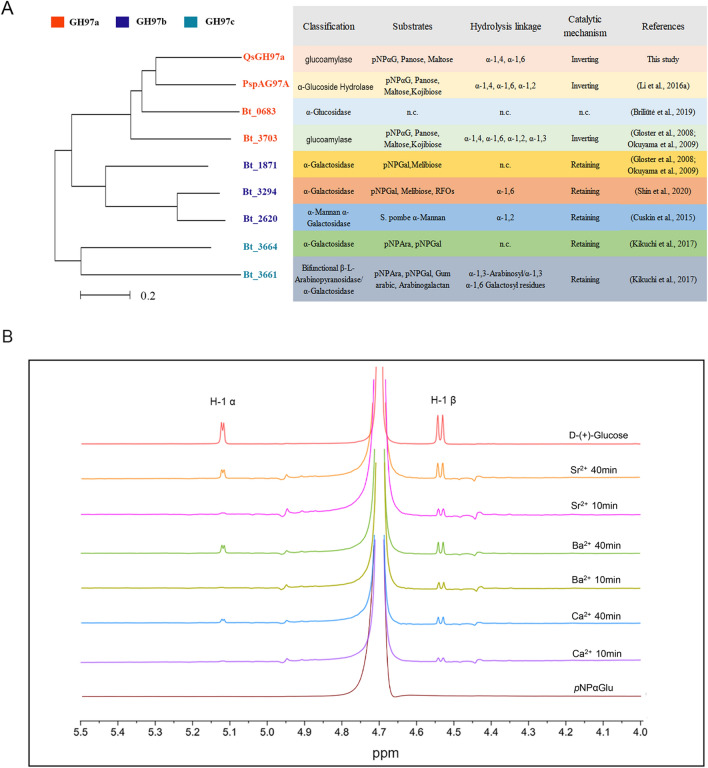
Characteristic of GH97 family members and the catalytic mechanism of QsGH97a. (**A**) Evolutionary relationship and characteristic comparison between QsGH97a and the characterized GH97 family enzymes. QsGH97a is classified into GH97a subfamily according to the mini evolutionary tree in the left picture; the information related to other characterized members is shown in the right picture, including classification, substrates, hydrolysis linkage and catalytic mechanism. GH97-derived enzymes are categorized into inverting enzyme and retaining enzyme based on the catalytic mechanism. ^a^n.c.: not characterized. (**B**) The result of ^1^H NMR spectroscopy. ^1^H NMR analysis showed QsGH97a has inverting mechanism to produce β-glucose. The peaks of H-1 α and H-1 β were shown in the group of d-(+)-Glucose, “Ca^2+^ 10 min” represents that the reaction mixture is measured at 40 °C for 10 min, “Ca^2+^ 40 min” exhibits that the reaction mixture is detected at 40 °C for 40 min. Ba^2+^-treated groups and Sr^2+^-treated groups are identical to Ca^2+^-treated groups.

A gene of 2037 bp, designated as *gsgh97a* encoding QsGH97a protein with 678 aa, was cloned from the whole-genome of strain *Qipengyuania seohaensis* SW-135. QsGH97a has a theoretical molecular weight of 75.57 kDa and pI of 4.97. The BLASTN search in the NCBI database displayed that *gsgh97a* showed the highest sequence similarity of 83.13% to the enzyme of GH97 from *Qipengyuania* sp. 1NDW3. The analysis result of multiple acid sequence alignments on characterized enzymes showed that QsGH97a shared the highest sequence identity of 50% with the α-glucoside hydrolase PspAG97A from *Pseudoalteromonas* sp. K8, followed by 39% identity with the glucoamylase SusB from *B. thetaiotaomicron*. There are three conserved catalytic residues (Glu 459, Glu 483, and Glu 378) in QsGH97a, which are equivalent to Glu 456, Glu 480, and Glu 377 in PspAG97A, respectively (Fig. 1A)^9^. In addition, two additional residues (Glu174 and Glu 477) may coordinate with Ca^2+^ based on the information of PspAG97A structure (Fig. 1A)^9^. Similar to other inverting enzymes, QsGH97a also contains conserved HHET and HE motifs. In contrast to inverting enzymes, the retaining enzymes, have a conserved Asp residue on the β4 chain that acts as a catalytic nucleophilic (Fig. 1B). On the other hand, according to the phylogenetic analysis, QsGH97a belongs to the enzymes from subfamily of GH97a (Fig. 2A and Supplementary Fig. S1).

### Purification and identification of QsGH97a

**Figure 3:**
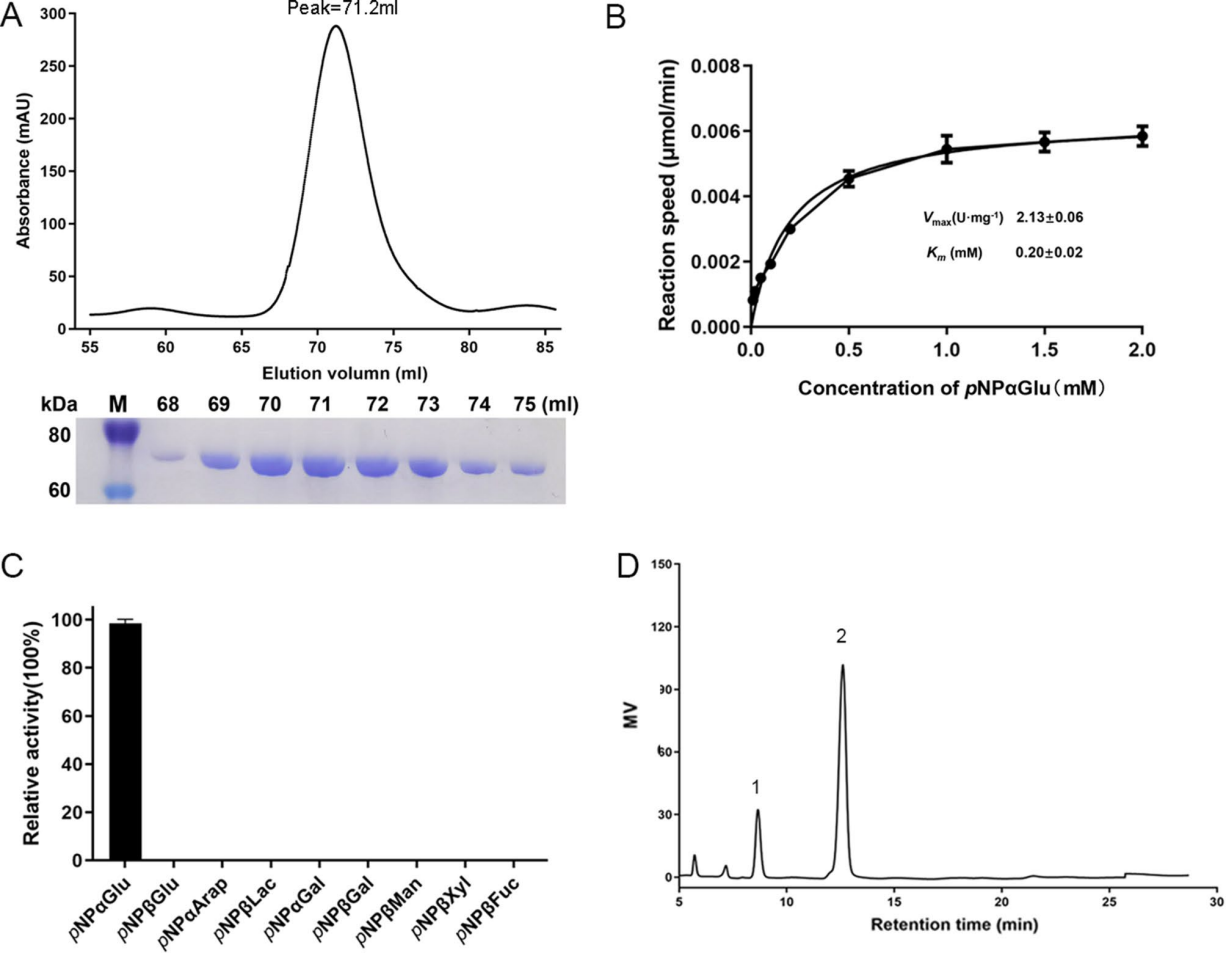
The SDS–PAGE result and the enzymatic activity analysis of QsGH97a. (**A**). Gel filtration chromatography of purified QsGH97a, the QsGH97a protein was eluted at the peak of 71.2 ml and SDS-PAGE result (bottom) of QsGH97a protein eluted from Superdex 200 16/600 column. Lanes from 1 to 9 were the molecular weight marker, and proteins with different elution volumes. Original blots/gels are presented in Supplementary Fig. S2B. (**B**) The kinetic curve on QsGH97a without addition metal. *v*_max_ = 2.13 ± 0.06 U mg^−1^; *K*_*M*_ = 0.20 ± 0.02 mM, (**C**) The results of hydrolysis activity of QsGH97a on different substrates. (**D**) The result of hydrolysis activity of QsGH97a on maltose, peak 1, glucose; peak 2, maltose.

Recombinant QsGH97a, with his-sumo tag, was successfully expressed in *E. coli* strain BL21 (DE3) plus and was purified by Ni^2+^-NTA affinity chromatography and gel-filtration chromatography. Its molecular mass was estimated to be 89.4 kDa. After cleavage of his-sumo tag with Ulp1 enzyme, the purified QsGH97a was obtained and observed as a single band in SDS-PAGE (Supplementary Fig. S2). The subunit molecular weight of QsGH97a was ~ 75.6 kDa, as shown by SDS-PAGE (Fig. 3A). Finally, 12 mg of target protein QsGH97a was harvested. In addition, the result of gel-filtration chromatography showed QsGH97a may be a dimer protein by comparison with the standard curve (Fig. 3A).

### Enzymatic characteristics of QsGH97a

**Figure 4:**
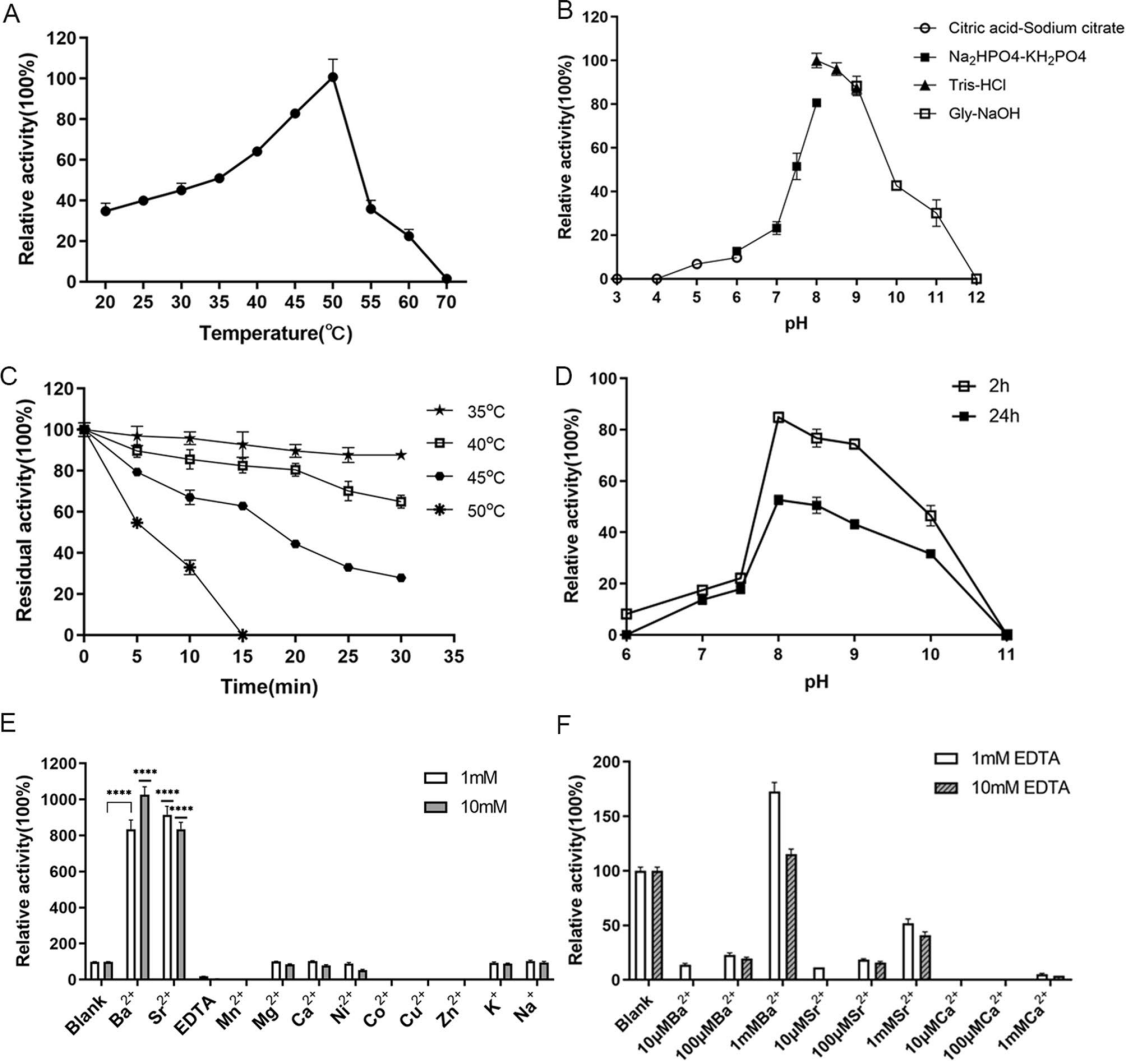
Enzymatic characterization of QsGH97a on *p*NPαGlu. (**A**) The activity of QsGH97a at various temperatures. The activity at 50 °C was regarded taken as 100%. (**B**) Effects of pH on enzyme activity. The activity at pH 8.0 was taken as 100%. (**C**) Thermal stability of QsGH97a incubated for 5–30 min at different temperatures. The activity incubated at 4 °C was taken as 100%. (**D**) pH stability of QsGH97a incubated for 2 h and 24 h at different pH values. The activity incubated at 4 °C was taken as 100%. (**E**) Effects of different metal ions on the QsGH97a activity. The activity without addition of metal ions was taken as 100%. ****Means a significant difference by comparison to blank group (*p* < 0.0001) (**F**) The results of the recovery experiment of the activity on EDTA-treated enzyme. The activity of untreated enzyme was taken as 100%.

The results of the hydrolysis activity of QsGH97a on different substrates were shown in Fig. 3C. QsGH97a exhibited the highest specific activity on *p*NPαGlu, and no activity on others. The activity of QsGH97a at various temperatures was shown in Fig. 4A. It was found that QsGH97a displayed the highest activity at 50 °C, moderate activity at 35–50 °C, and low activity at 55–70 °C, suggesting that the optimal temperature for QsGH97a was 50 °C. The temperature stability experiment revealed that QsGH97a could retain above 50% activity at 35–40 °C, low activity at 45 °C, but was rapidly inactivated at 50 °C after 30 min incubation (Fig. 4C). The enzyme activity in the optimal reaction condition was defined as 100%. The process of enzymatic reaction under various temperatures ranging from 35 to 50 °C was shown in Supplementary Fig. S3. The results of QsGH97a activity at various pH values showed that QsGH97a exhibited an optimal activity at pH 8.0 (Fig. 4B) and a rapid loss activity in the environment of strong acid (pH 3.0–pH 5.0) and alkali (pH 12.0). Within the range of pH 7.5–pH 9.0, QsGH97a maintained more than 50% of activity at the optimum pH. QsGH97a was relatively stable in alkaline conditions with the range from pH 8.0 to pH 10.0 at 4 °C after 2 h incubation. Besides, it could still retain greater than 50% activity for 24 h in pH 8.0–pH 8.5 at 4 °C (Fig. 4D).

**Figure 5:**
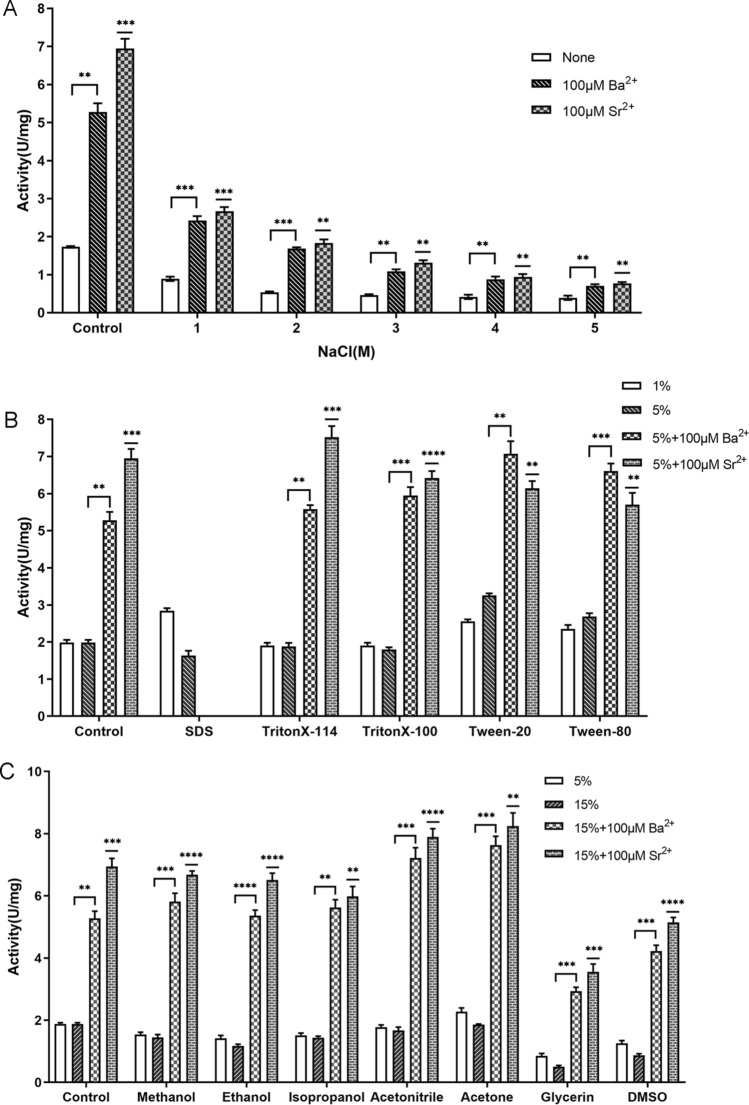
Enzymatic characterization of QsGH97a on *p*NPαGlu without and with addition of Ba^2+^ and Sr^2+^. (**A**) Effects of 100 μM Ba^2+^or Sr^2+^ on QsGH97a activity in different NaCl concentrations (1 M, 2 M, 3 M, 4 M, 5 M). The activity of control group was detected without addition of NaCl (**B**) Effects of 100 μM Ba^2+^ and Sr^2+^ on QsGH97a activity in 5% various detergents. The activity of control group was detected without addition of detergents. (**C**) Effects of Ba^2+^ and Sr^2+^ on QsGH97a activity in 15% various organic solvents. The activity of control group was detected without addition of organic solvents. In figure, * means a significant difference by comparison to control group for each group (*p* < 0.05), ** means a significant difference of *p* < 0.01, *** means a significant difference of *p* < 0.001, **** means a significant difference of *p* < 0.0001.

In addition, we evaluated the effect of metal ion on the enzyme activity by adding 1 mM or 10 mM different metal ions. The results were shown in Fig. 4E, the enzyme activity without extra metal ions was considered as 100%. It was shown that Ba^2+^ or Sr^2+^ could strongly stimulate QsGH97a activity by 8–10 folds, which was statistically significantly different compared to the blank group (*p* < 0.0001). While EDTA, Co^2+^, Cu^2+^, Zn^2+^, or Mn^2+^ almost entirely abolished the activity. By adding Ba^2+^ or Sr^2+^, the inhibitory impact of EDTA on the enzyme could be eliminated (Fig. 4F). The enzyme activity increased steadily as the concentration of Ba^2+^ or Sr^2+^ increased from 20 µM to 20 mM or 20 µM to 5 mM, respectively, and the enzyme activity decreased when the Sr^2+^ concentration exceeded 5 mM (Supplementary Fig. S4). The QsGH97a activity was not significantly affected with addition of Mg^2+^, Ca^2+^, Ni^2+^, K^+^, or Na^+^. Furthermore, the effects of different NaCl concentrations on the QsGH97a activity were examined (Fig. 5A). QsGH97a showed 30% enzymatic activity in the presence of 1 M NaCl, and could maintain approximately 20% relative activity in 5 M NaCl. The majority of detergents had a little impact on the catalytic activity of QsGH97a (Fig. 5B). Interestingly, addition of 1% SDS, 5% Tween20, or 5% Tween80 could slightly enhance catalytic activity. Most organic solvents exerted a slightly negative effect on enzymatic activity (about 50–70%), such as methanol, ethanol, and isopropanol (Fig. 5C). However, the catalytic activity of QsGH97a can be greatly depressed in the presence of 15% glycerin or 15% DMSO.

### Kinetic analysis of QsGH97a without and with addition of Ba^2+^ or Sr^2+^

The results above revealed that the QsGH97a activity could be significantly improved with the presence of Ba^2+^ and Sr^2+^. To confirm the effects of Ba^2+^ and Sr^2+^, the kinetic parameters were analyzed in the absence or presence of Ba^2+^ or Sr^2+^ at concentrations of 100 μM and 1 mM (Supplementary Table S2). The enzymatic activity of QsGH97a was 2.13 U mg^−1^ in the absence of Ba^2+^ or Sr^2+^, and the *K*_*m*_ and *k*_cat_ were 0.20 mM and 5.40 S^−1^, respectively (Fig. 3B, and Supplementary Table S2). With the addition of Ba^2+^ or Sr^2+^, the kinetic values were drastically improved. The enzymatic activity values were increased to 11.41 U mg^−1^ and 12.97 U mg^−1^ with addition of 1 mM Ba^2+^ and 1 mM Sr^2+^, respectively. However, the apparent affinity of the enzyme for the substrate was reduced, and the *K*_*m*_ values were 0.48 mM and 0.36 mM, respectively.

### Effects of Ba^2+^ or Sr^2+^ on enzyme activity and stability

**Figure 6:**
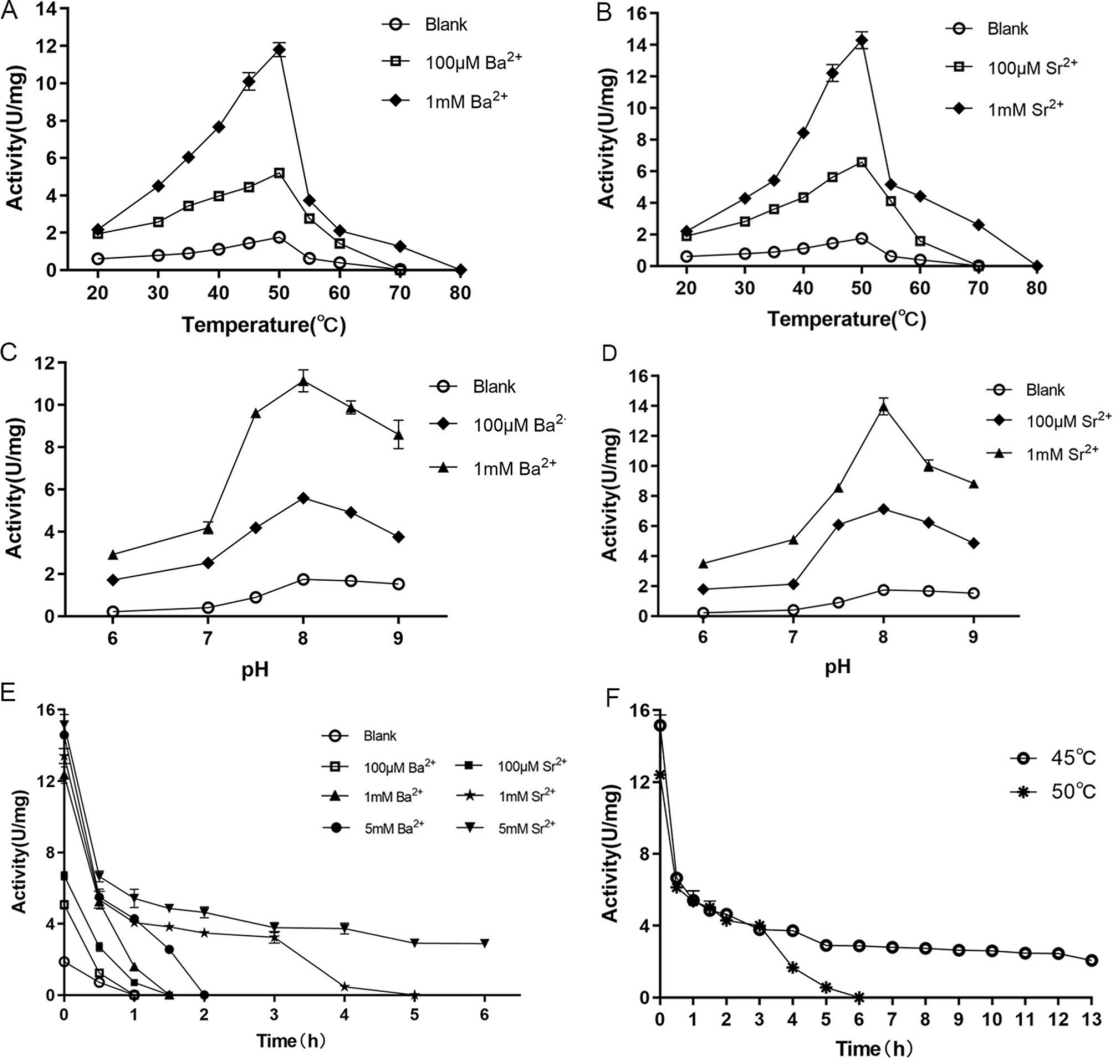
Effects of temperature and pH on QsGH97a activity and stability with addition of Ba^2+^ and Sr^2+^. (**A**) The effect of different concentrations Ba^2+^ on the optimum temperature of QsGH97a. Ba^2+^ was not added in the blank group. (**B**) The effect of different concentrations Sr^2+^ on the optimum temperature of QsGH97a. Sr^2+^ was not added in the blank group. (**C**) The effect of different concentrations Ba^2+^ on the optimum pH of QsGH97a. Ba^2+^ was not added in the blank group. (**D**) The effect of different concentrations Sr^2+^ on the optimum pH of QsGH97a. Sr^2+^ was not added in the blank group. (**E**) The effect of different concentrations Ba^2+^ and Sr^2+^ on the thermal stability of QsGH97a at 45 °C. The blank group was incubated at 4 °C without addition of Ba^2+^ and Sr^2+^. (**F**) The effect of 5 mM Sr^2+^ and 10 mM Sr^2+^on the thermal stability of QsGH97a at 45 °C and 50 °C, respectively. The blank group was incubated at 4 °C without addition of Sr^2+^. The above substrate was *p*NPαGlu.

The effects of Ba^2+^ or Sr^2+^ on enzyme activity and stability were also determined at various temperature levels. As illustrated in Fig. 6A,B, the activity was generally elevated at different concentrations of Ba^2+^ or Sr^2+^, especially at 1 mM Sr^2+^. QsGH97a exhibited an optimal activity at 50 °C. The enzyme thermostability in 45 °C was also greatly improved in the presence of Ba^2+^ or Sr^2+^ (Fig. 6E). The higher the concentration of Sr^2+^, the more obvious the thermostability. Enzymatic activity of QsGH97a could still maintain higher than 100% at 45 °C or 50 °C after 13 h and 4 h incubation with addition of 5 mM and 10 mM Sr^2+^ (Fig. 6F). In addition, at different concentrations of Ba^2+^ or Sr^2+^_,_ the optimum pH was still 8.0 (Fig. 6C,D).

We then evaluated the enzymatic activity with addition of NaCl, detergents, and organic solvents in the presence of 100 μM Ba^2+^ or Sr^2+^. The addition of Ba^2+^ or Sr^2+^ considerably improved the salt tolerance of QsGH97a, and the maximum activity of the enzyme in 5 M NaCl was about 0.8 U mg^−1^ (Fig. 5A). The activity of QsGH97a in the presence of detergents and organic solvents was also greatly elevated with Ba^2+^ or Sr^2+^ addition; however, the activity toward SDS was not determined since the samples precipitated. When treated with 5% detergent or 15% organic solvent, the tolerance of enzyme to them also could be elevated by ~ fourfold in the presence of Ba^2+^ or Sr^2+^. The enzyme was more tolerant to glycerin and DMSO than the control group (Fig. 5B,C). Statistical analysis showed significant differences both Ba^2+^-treated and Sr^2+^-treated groups compared to the control group (*p* < 0.05).

### Analysis of enzymatic transglycosylation activity

Since the CAZy database annotation shows that QsGH97a is an α-glucosidase, some α-glucosidases have a transglycosidic function which allows them to link free-glucose after hydrolysis with α-1, 6 linkages to form functional isomaltose–oligosaccharides^16^. HPLC was used to analyze the transglycoside activity of QsGH97a on maltose. The results showed that the enzyme only hydrolyzed maltose to glucose without the formation of transglucosylated products such as panose, isomaltose, or isomaltotriose (Fig. 3D). Subsequent results discovered that QsGH97a was capable of hydrolyzing panose, isomaltose, and isomaltotriose to produce glucose (Supplementary Fig. S6B–D). Compared with the optimal substrate *p*NPαGlu, the hydrolysis activity of the enzyme to maltose, panose, isomaltose, and isomaltotriose was 8.7%, 12.7%, 21.8%, and 17.8%, respectively (Supplementary Table S1).

### Anomeric form of QsGH97a hydrolytic product

In order to explore the specific catalytic mechanism, the anomeric form of hydrolysis product of *p*NPαGlu by QsGH97a with addition of 1 mM Ca^2+^, Ba^2+^ or Sr^2+^ were determined by ^1^H NMR. The accumulation of β-glucose was observed after 10 min of reaction in every experimental groups. When the reaction time reached 40 min, α-glucose also appeared (Fig. 2B). The formation of α-glucose can be explained by spontaneous mutation of the hydrolysis product, β-glucose^8^. The results showed that the hydrolysis reaction of BtGH97a adopted an inverting mechanism to generate β-d-glucoses with addition of Ca^2+^, Ba^2+^ or Sr^2+^. This discovery is consistent with the sequence analysis of QsGH97a, which contains Glu residue (Glu 378) that acts as a catalytic base on β-strands 3 and 5, essential for inverting mechanism.

### The predicted three-dimensional structure of QsGH97a

**Figure 7:**
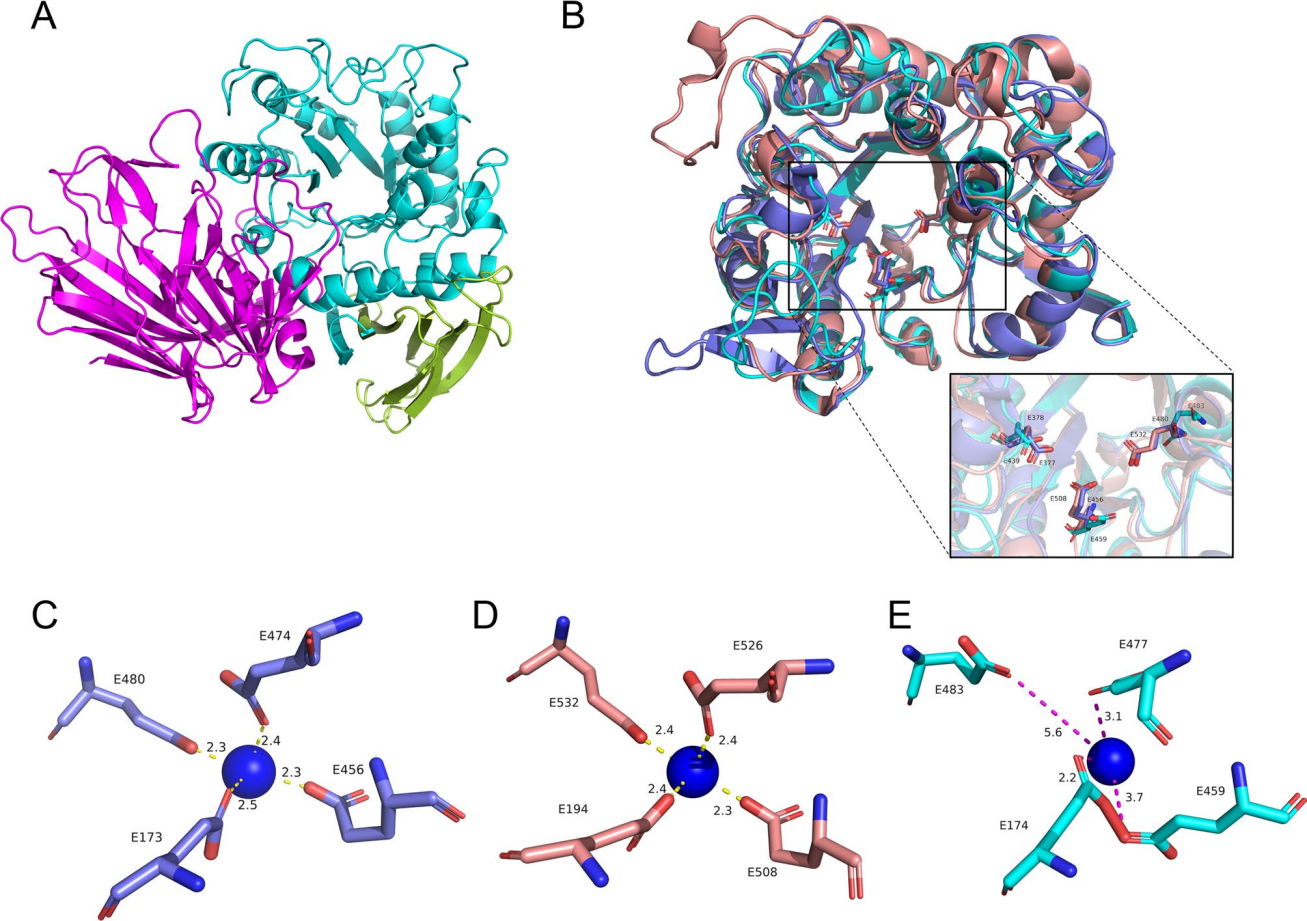
The predicted three-dimensional structure of QsGH97a. (**A**) The forecast overall structure of QsGH97a, the N-terminal domain is shown in magenta, the core (β/α)8domain in cyan, and the C-terminal domain in split pea. (**B**) The alignment of predicted QsGH97a (cyan) catalytic domain with PspAG97A (PDB ID: 5HQ4) (slate) and SusB (PDB ID: 2JKA) (salmon). The Glu residues in catalytic sites were marked. (**C**–**E**) The distance between Ca^2+^ and interacting Glu residues. The blue ball represents calcium ion; picture (**C**) is QsGH97a; picture (**D**) is 5HQ4; and picture (**E**) is 2JKA.

The predicted three-dimensional structure of QsGH97a was exhibited in Fig. 7A. QsGH97a also comprises classical three domains: an N-terminal β-sandwich domain (residues Ala21–Asp277), a canonical (β/α)_8_ barrel (residues Val278–Glu586), and a C-terminal β-sheet domain (residues Phe587–Pro676), which has been characterized in GH97^7,9^. Pymol software^17^ was used to compare the position of the catalytic residues among QsGH97a, PspAG97A (PDB ID: 5HQ4), and SusB (PDB ID: 2JKA). It was observed that the catalytic residues of QsGH97a were not highly conserved relative to the other two members from GH97 (Fig. 7B). At the same time, the pattern diagrams of the interaction between the Ca^2+^ and its residues in the active site of different enzymes were compared. The predicted Glu483 residue of QsGH97a that coordinates with Ca^2+^ is far from calcium ions with 5.6 Å (Fig. 7C). The distance between the Ca^2+^ and the coordinating residues in the PspAG97A structure (PDB ID: 5HQ4) ranges from 2.3 to 2.5 Å, which is 2.3–2.4 Å in SusB structure (PDB ID: 2JKA) (Fig. 7D,E)^9^.

## Discussion

Glucoamylases are found throughout the ecosystem, the majority of which are produced by microbes. Among them, enzymes from marine bacterium generally exhibit innovative qualities including cold adaptability, salt tolerance, and metal resistance, and thus they are widely used in industry^18–20^. In this work, we characterized a glucoamylase named QsGH97a from a deep-sea bacterium *Qipengyuania seohaensis* SW-135. Although the CAZy database and the bioinformatic analysis show that QsGH97a is an α-glucosidase of GH97. The substrate specificity showed that QsGH97a can strongly hydrolyze α-1,4-glucoside linkage and weakly hydrolyze α-1,6-glucosidic linkage, which was similar to the characteristic of glucoamylases. α-Glucosidases (EC 3.2.1.20, α-d-glucoside glucohydrolase) cleave α-1,4-glycosidic linkages from the non-reducing end of oligosaccharide substrates to produce free α-glucose via a retaining mechanism^8,21^. While Glucoamylases utilize a classic inverting mechanism that hydrolyzes α-d-glucose from the nonreducing terminal of α-glucan chain to release β-glucose^22^. Subsequent NMR experiments discovered that QsGH97a employs an inverting mechanism to produce β-glucose. These characteristics revealed that QsGH97a function as a glucoamylase that can cleave the α-1,4-glycosidic bonds and α-1,6-glycosidic bonds, not an α-glucosidase. SusB and PspAG97A from the GH97 family are different from linkage affinity, despite that they can also act on α-1,6-glucosidic linkage, and can cleavage α-1,2-glycosidic linkage, etc.^14^ (Fig. 2A and Supplementary Table S3). PspAG97A showed higher catalytic activity towards α-1,6-linked substrates, and high catalytic efficiency on dextran composed of α-1,6/α-1,3/α-1,4-linkage^14^. SusB also hydrolyzed α-1,2 and α-1,3- linkage in glucobioses and exhibited the highest activity on *p*NPαGlu. The comparison result showed that the sizes of substrate-binding pockets of the three enzymes were different, which may explain why they have varying affinities for different substrates (Supplementary Fig. S7). Therefore, QsGH97a is a novel glucoamylase with special biochemical characteristics in the GH97 family.

The optimal temperature of QsGH97a was 50℃, consistent with glucoamylase from *Corallococcus* sp. strain EGB and higher than that from GH97^8,23^ (Supplementary Table S3). The optimum pH for the majority of known glucoamylases varies from 4.5 to 7.0^24–26^. QsGH97a exhibited the optimal activity at pH 8.0 and showed higher activity in alkaline environment with addition of Ba^2+^ or Sr^2+^. The alkophilic and alkali-resistant properties of QsGH97a were similar to enzymes derived from other deep-sea organisms^27,28^. These properties enable it to be employed in industrial applications that require an alkaline environment, such as the production of detergents and papermaking. Furthermore, QsGH97a showed greater resistance to high concentrations of salt, detergents, and organic solvents in the presence of metal ions Ba^2+^ or Sr^2+^. A novel esterase and an α-Glucosidase were also characterized in *Qipengyuania seohaensis* SW-135, which are highly alkaliphilic, halotolerant, and resistant to detergents and organic solvents^29,30^. The research has confirmed that this bacterium was a mildly halophilic bacterium and did not grow in a medium without NaCl or with addition of > 9% NaCl^28^. This could explain why the enzymes extracted from this strain were salt-tolerant to a certain extent. In the future, we will identify more enzymes from this bacterium, which can be applied to industries that require alkali and salt-resistance enzymes. Further, we will broaden the application range of enzymes with new characteristics and provide a technology for industrial modification of enzyme properties.

Glucoamylases exhibit different responses to diverse metal ions^23^. Ca^2+^ is usually an important metal ion involved in most glucoamylase reactions and maintaining the enzyme stability^31,32^. As mentioned before, enzymes from GH97 have Ca^2+^ in their catalytic centers, which can stabilize the activity of enzymes. *Escherichia coli* β-galactosidase (lacZ) from GH2 is Mg^2+^ dependent and α-mannosidases from GH38 and GH47 are Zn^2+^ and Ca^2+^ dependent, respectively^7,33,34^. The bulk of glucoamylases from GH15 are Ca^2+^ dependent. Interestingly, QsGH97a activity can be strongly stimulated by metal ions such as Ba^2+^ or Sr^2+^, and the activity could be increased by 8–13 folds. QsGH97a did not exhibit a strong dependence on Ca^2+^, and 1 mM Ca^2+^ could not restore enzyme activity of EDTA-inactivated QsGH97a. On the other hand, 1 mM Ba^2+^ or 1 mM Sr^2+^ addition allowed EDTA-treated QsGH97a to recover 50–100% activity (Fig. 4F). It was suggested that the removal of Ba^2+^ and Sr^2+^ may trigger a conformational change at the substrate binding sites, which is not conducive to a complete revival of activity. We propose that the catalytic center may be coordinated by Ba^2+^ and Sr^2+^ rather than Ca^2+^. In order to confirm our hypotheses, we further detected the content of metal ions in the purified enzymes using ICP-OES after adding Ba^2+^ and Sr^2+^ to *E. coli* culture. We discovered a relatively high proportion of Sr^2+^ (67–192%), and a low content of Ba^2+^ (~ 9%), we speculated that QsGH97a bond Sr^2+^ more preferentially than Ba^2+^ (Supplementary Fig. S5). Enzymes with high content of Sr^2+^ were more active (data not shown). QsGH97a adopt inverting catalytic mechanism in the presence of Ba^2+^ or Sr^2+^ based on NMR analysis, indicating that strontium and barium enzymes possess the same mechanism. Furthermore, by predicting its three-dimensional structure and comparing the difference between the residues of QsGH97a and other enzymes that interact with Ca^2+^, it was shown that the predicted Glu483 residue is farther away from Ca^2+^. We speculated that these residues are not coordinated with Ca^2+^, but supposedly Ba^2+^ or Sr^2+^. The ionic radius of Ba^2+^ or Sr^2+^ is slightly greater than that of Ca^2+^, which may be more suitable for the steric property of the catalytic sites. Ca^2+^, Ba^2+^ and Sr^2+^ are alkaline earth metals with similar biological characteristics. Studies have shown that Ba^2+^ and Sr^2+^ can replace Ca^2+^ in the methanol dehydrogenase catalytic active center^35,36^. Perhaps GH97 also contains this type of intriguing metal ion substitution mechanism. In seawater, the content of Sr is relatively high^37^, so it is conceivable that QsGH97a can utilize Sr^2+^. Alternatively, with the presence of Sr^2+^, thermostability and optimal temperature of enzyme was enhanced, and the higher the concentration of Sr^2+^, the more significant the effect. That is, not only QsGH97a activity but also its thermostability may be dependent on Sr^2+^. Enzymes with fine thermal stability can be widely used for industrial production.

Thus, GH97 is a unique GH family, with an unclear catalytic mechanism but a potential for industrial application. We characterized a novel enzyme QsGH97a, in which the addition of Ba^2+^ and Sr^2+^ can significantly promote the enzymatic reaction process, and its thermal stability. Moreover, QsGH97a is resistant to organic solvents and detergents and can maintain activity in high-salt environments, which makes it industrial-promising. However, we lack accurate knowledge of protein structure to explain the peculiarities of the catalytic mechanism. In the next step, we will focus on the specific mechanisms of enzyme binding to different substrates and coordination with metal ions, so as to enrich the functional research of the GH97 family and further explore its potential applications in the industry.

## Materials and methods

### Sequence analysis and prediction of the three-dimensional structure

The information of nucleotide and amino acid sequence alignments was obtained by BLAST server (http://blast.ncbi.nlm.nih.gov/Blast.cgi). The deduced amino acid sequence was analyzed by ExPASy Proteomics Server (http://www.expasy.ch/tools/). Multiple alignment analysis of amino acid sequences was performed by using Clustal Omega (http://www.ebi.ac.uk/Tools/msa/clustalo/)^38^ and ESpript v.3.0 server^39^. The phylogenetic tree was constructed by MEGA (Molecular Evolutionary Genetics Analysis) v.10.1 software^40^ using Neighbor-Joining algorithm and Bootstrap analysis, which decorated by the iTOL server (https://itol.embl.de/). The three-dimensional structure of enzyme was forecasted via AlphaFold2 which is widely regarded as a trustworthy technique for protein structure prediction^41^. The structure diagrams were created using the Pymol 3.8 software^17^.

### Bacterial strains and vector

*Qipengyuania seohaensis* SW-135 (NCBI Taxonomy database, accession number:266951), a member of *Erythrobacteraceae* family, was isolated from inter sediments in the Yellow Sea in Korea^42^. The plasmid pSMT3 with a his-sumo tag was used for gene cloning and expression^43^. *E. coli* DH5α strain (Transgen, China) was used in gene clone for *qsgh97a*. *E. coli* BL21 (DE3) plus strain (Transgen, China) was used as the host for protein expression. *E. coli* BL21 (DE3) plus was cultured at 37 °C in LB liquid medium and LB agar medium added with 1.5% (w/v) agar.

### Gene cloning of *qsgh97a*

Primers used to amplify *qsgh97a* (GenBank database, accession number: CP024920.1) include: forward primer (5′-GGCGGATGATCCGCCATATCGCCCTCTTCATC-3′), and reverse primer (5′-AAACTCGAGTCACCCCTGCGGCACGAACTCG-3′). The cloned target gene fragment and pSMT3 vector were subjected to double restriction enzyme digestion with *Bam*HI and *Xho*I, respectively. The fragment and vector were connected by T4 DNA ligase with molar ratio of 7:1. The restriction enzymes and T4 DNA ligase were purchased from NEB company (The United States). The obtained recombinant vectors transformed into *E. coli* DH5α were spread on LB agar plates containing 50 μg/mL kanamycin. True positive clones were screened using PCR technique and then stored at − 80 °C once their sequence accuracy was verified via sequencing.

### Expression of the *qsgh97a* in *E. coli*

In order to express the *qsgh97a*, the first step was to extract the recombinant plasmid in *E. coli* DH5α. The recombinant plasmid was transformed into *E. coli* BL21 (DE3) plus for protein expression and grown at 37 °C in LB plate with chloramphenicol (34 μg/mL) and kanamycin (50 μg/mL). Constructs expressing *qsgh97a* were grown in 500 mL LB liquid medium at 37 °C with shaking at 200 rpm. When the OD600 reached 0.8, isopropyl β-d-thiogalactoside with final concentration of 0.5 mM was added to cells followed by incubation at 16 °C for 20 h.

### Purification and identification of glucoamylase QSGH97a

The purification protocol was similar to the previous report with minor revisions^44^. *E. coli* cells were collected by centrifugation at 5000 rpm for 15 min at 4 °C. Then, the pellets were resuspended in buffer A (50 mM Tris–HCl, pH 8.0, 500 mM NaCl, 10 mM imidazole, 1% glycerol, 1 mM DTT) containing 0.2 mM PMSF (Phenylmethylsulfonyl fluoride). Then resuspended cells were disrupted by ultrasound and centrifuged at 12,000 rpm for 15 min at 4 °C. The supernatant was then added to Ni–NTA column (QIAGEN, Germany) pre-equilibrated with buffer A. Buffer A and buffer B (50 mM Tris–HCl, pH 8.0, 500 mM NaCl, 20 mM imidazole, 1% glycerol, 1 mM DTT) was used to remove unbound and unspecifically bounded proteins, respectively. Finally, the his-tag protein was washed by buffer C (50 mM Tris–HCl, pH 8.0, 500 mM NaCl, 250 mM imidazole, 1% glycerol, 1 mM DTT). The protein samples were analyzed by 12.5% SDS-PAGE. Then, the purified proteins were concentrated and desalted. Ulp1 enzyme was added to the concentrated sample and incubated overnight at 4 °C in order to cleave the his-sumo tag^45^. The sample was added to Ni–NTA column pre-equilibrated with buffer A. The effluent sample was collected to concentrate and perform the further purified experiment. At last, the concentration was evaluated by the method of Bradford with bovine serum albumin (BSA)^46^. Additionally, the protein without his-sumo tag was further purified by gel-filtration chromatography Superdex 200 16/600 column (GE, USA) and identify the molecular weight of protein. The enzyme solution was replaced with buffer D (20 mM Tris–HCl, pH 7.4, 100 mM NaCl, 1 mM DTT).

### Enzyme activity assay

The enzymatic activity of QsGH97a was measured by *p*-nitrophenol method as described as previously^47^. A reaction mixture (100 μl) containing 0.03 mg enzyme, 2 mM *p*NPαGlu (Shanghai yuanye, China), and 20 mM Tris–HCl buffer (pH 8.0) was incubated at 50 °C for 1 min in linear period (determined at 405 nm). The reaction speed in linear period reflects the enzymatic activity. One unit of enzymatic activity is defined as the amount of enzyme that catalyzes the production of 1 μmol *p*NP per minute. All experiments were carried out with three biological replicates.

### Substrate specificity

Different substrates (Shanghai yuanye, China) were measured including *p*NPβGlu (*p*-Nitrophenyl-β-d-glucopyranoside), *p*NPαGlu (*p*-Nitrophenyl-α-d-glucopyranoside), *p*NPαArap (*p*-Nitrophenyl-α-l-arabinopyranoside), *p*NPβLac (*p*-Nitrophenyl-β-d-lactopyranoside), *p*NPβGal (*p*-Nitrophenyl-β-d-galactopyranoside), *p*NPβMan (*p*-Nitrophenyl-β-d-mannopyranoside), *p*NPβXyl (*p*-Nitrophenyl-β-d-xylopyranoside), *p*NPβFuc (*p*-Nitrophenyl-β-d-fucopyranoside), *p*NPβCel (*p*-Nitrophenyl-β-d-cellobioside). At last, enzymatic activity of each group was compared. The highest enzymatic activity was regarded as 100%^48^.

### Effects of temperature and pH on activity and stability of QsGH97a

The optimum temperature and optimum pH were obtained by measuring the activities of QsGH97a in different temperatures and pH values, respectively. The activities of QsGH97a were measured at different temperatures from 20 to 70 °C. The reaction mixture was mentioned above. The temperature stability was measured by incubating the enzyme in 20 mM Tris–HCl (pH 8.0) at 35–50 °C for 5 to 30 min. The activity of the untreated group was taken as 100% residual activity^49^. To determine optimum pH values, standard buffer were used as follows: 20 mM citric acid buffer (pH 3.0–6.0), 20 mM phosphate buffer (pH 6.0–8.0), 20 mM Tris–HCl buffer (pH 7.5–9.0) and 20 mM Gly-NaOH buffer (pH 9.0–12.0). The pH stability was tested by incubating the enzyme in pH 3.0 to pH 12.0 at 4 °C for 2 h and 24 h. Finally, the residual activity of each group was measured at the optimum temperature. The activity of the untreated group at optimum temperature and pH was taken as 100%^21^.

### Effects of various metal ions and NaCl concentration on enzyme activity

Enzyme activities were evaluated with addition of 1 mM or 10 mM solutions of different metal ions (EDTA, MnCl_2_·4H_2_O, BaCl_2_·2H_2_O, SrCl_2_·6H_2_O, MgCl_2_·6H_2_O, CaCl_2_, NiCl_2_·6H_2_O, CoCl_2_·6H_2_O, CuCl_2_·2H_2_O, ZnSO_4_·7H_2_O, KCl, and NaCl). The effects of NaCl on enzyme activity were measured by adding different concentration solutions (5 M, 4 M, 3 M, 2 M, 1 M) of NaCl. The reaction mixture was mentioned above. The enzyme activity in the untreated group was taken as 100%.

### Effects of detergents and organic solvents on enzyme activity

To evaluate the effect of detergents (SDS, Triton X-114, Triton X-100, Tween 20, and Tween 80) on the enzyme activity, the enzyme was incubated with each detergent at 1% v/v and 5% v/v concentration in 20 mM Tris–HCl buffer (pH 8.0) at 50 °C. Similarly, 5% v/v and 15% v/v organic solvents were used to measure enzyme activity including methanol, ethanol, isopropanol, acetone, acetonitrile, glycerin, and dimethyl sulfoxide (DMSO).

### Kinetic determinations of enzyme without and with addition of Ba^2+^ or Sr^2+^

The kinetic parameters of QsGH97a were determined at the optimum temperature and pH. The 100 μl reaction mixture included 0.03 mg enzyme, 20 mM Tris–HCl buffer (pH 8.0) and *p*NPαGlu of different concentrations (0.01 mM, 0.02 mM, 0.05 mM, 0.1 mM, 0.2 mM, 0.5 mM, 1.0 mM, 1.5 mM, 2.0 mM). The standard curve of *p*NP (Macklin, China) was used to calculate the amount of *p*NP released. The reaction rate of each group was measured at 50 °C and pH 8.0, and the *K*_*m*_ value and *V*_max_ were counted according to the Michaelis–Menten equation, with GraphPad Software^44^.

### Effects of Ba^2+^ or Sr^2+^ on enzyme tolerance of NaCl, detergents and organic solvents

The steps for detecting enzyme activity were as described above, and the conditions of the experimental groups contained NaCl with concentrations of 1 M, 2 M, 3 M, 4 M, 5 M, 5% v/v detergents, and 15% v/v organic solvents. In addition, the experimental groups were added with 100 μM metal ions, and the control group was not added with any additional substances. Each group had three replicates.

### Statistical analysis

The differences among Ba^2+^-treated or Sr^2+^-treated and control groups were analyzed using two-way ANOVA analysis, followed by Tukey’s test Multiple Comparison (α = 0.05) to further compare the differences^50^. Statistical significance was defined as a *p*-value < 0.05 for differences in means. GraphPad software was used for the statistical analysis described above.

### Analysis of High Performance Liquid Chromatography

The reaction mixture consisted of 500 µl of 15% (w/v) maltose solution in 20 mM Tris–HCl buffer (pH 8.0) and 0.4 mg of enzyme, which was incubated at 45 °C for 12 h. After the reaction, the reaction mixture was incubated at 100 °C for 10 min to denature and inactivate the enzyme. Subsequently, the mixture was centrifuged at 12,000 rpm for 10 min. Finally, the supernatant was diluted 10 times by mobile phase and analyzed by HPLC^51^. The analysis was detected by LC-20AT (Shimadzu) equipped with Hypersil NH_2_ column (4.6 × 250 mm) (Thermo Fisher Scientific). The mobile phase was composed of 72% acetonitrile and 28% water and the flow rate is 0.8 ml/min. A refractive index detector RID-10A (Shimadzu) was used to check the carbohydrates.

### Metal content analysis

The detection procedures and methods refer to previous studies^44,52^. The samples were purified enzymes which were expressed in 50 μM and 100 μM Ba^2+^, or Sr^2+^ environment. 188–488 μl of samples (0.41–1.06 mg/ml) were deconstructed with 10% nitric acid at 90 °C for 1 h. Then purified enzymes were diluted to 5 ml with MilliQ water. Subsequently, the processed samples were sent to the laboratory (Chemistry and Chemicl Engineering of Central South University) for detection of metal ions by using ICP-OES (PerkinElmer Avio 500).

### Nuclear magnetic resonance

^1^H NMR spectra was used to detect the conformation of the hydrolyzates^7^. The mixture of samples was 500 μl, the experimental group contained 30 μg enzyme, water-soluble substrate with the final concentration of 18 mM, and 1 mM Ca^2+^, Ba^2+^, or Sr^2+^. Assays were run at 40 °C for 10 min and 40 min. The standard was 5 mg d-(+)-glucose; and the control group included water-soluble substrate without addition of enzyme. Then 100 μl D_2_O was added to each group before NMR spectra. Finally, all the samples were sent to the Institute for Advanced Research in Central South University for testing hydrolyzates by using Bruker 600 MHz spectrometer (AVANCE III 600 M, Switzerland).

## Conclusion

In summary, a novel glucoamylase QsGH97a from GH 97 was cloned and expressed successfully from *Qipengyuania seohaensis* SW-135, and the enzymatic properties were characterized in detail. QsGH97a shows the maximal activity at 50 °C and pH 8.0. The biochemical characterization shows that QsGH97a exhibits good stability in an alkaline environment with addition of Ba^2+^ or Sr^2+^. Additionally, the catalytical activity and thermostability can be strongly enhanced with addition of Ba^2+^ or Sr^2+^, which has been established for the first time in glucoamylases. The enzymatic properties of QsGH97a will deepen our understanding of the catalytic mechanism of the GH97 family and set the stage for the industrial application of glucoamylase.

## Supplementary Information

Supplementary Information.

## Data Availability

The ^1^H NMR datasets generated and/or analyzed during the current study are stored in the Figshare repository. https://doi.org/10.6084/m9.figshare.21825162.
