# Supplementary material for: The effect of barium and strontium on activity of glucoamylase QsGH97a from Qipengyuania seohaensis SW-135
Source: Sci Rep. 2023 Apr 10;13:5840. doi: 10.1038/s41598-023-32161-y (PMC10086023; doi:10.1038/s41598-023-32161-y)
Supplement: Supplementary file 1 — Supplementary Information. [file 41598_2023_32161_MOESM1_ESM.pdf]

Table S1. The hydrolysis activity of QsGH97a on various substrates.

Substrate	Relative activity
*p*NPαGlu	100.00%
*p*NPβGlu	0.00%
*p*NPαArap	0.00%
*p*NPβLac	0.00%
*p*NPαGal	0.00%
*p*NPβGal	0.00%
*p*NPβMan	0.00%
*p*NPβXyl	0.00%
*p*NPβFuc	0.00%
*p*NPβCel	0.00%
Maltose	8.72%
Panose	12.66%
Isomaltose	21.79%
Isomaltotriose	17.78%

Table S2. Kinetic determinations of QsGH97a without and with addition of Ba^{2+} or Sr^{2+}.

		Sr^{2+}		Ba^{2+}	
	None	100μM	1mM	100μM	1mM
K_m (mM)	0.20±0.02	0.47±0.05	0.36±0.04	0.35±0.02	0.48±0.03
V_{max} (U.mg^{-1})	2.13±0.06	6.68±0.25	12.97±0.40	5.12±0.11	11.41±0.28
k_{cat} (S^{-1})	5.40±0.15	16.83±0.63	32.16±0.99	12.91±0.28	28.74±0.70
k_{eff} (S^{-1}mM^{-1})	27.3±3.50	36.02±5.13	90.12±7.82	37.27±2.92	60.10±5.22

[a]k_{eff} is defined as k_{cat}/K_M

Table S3. Comparison of the functions and properties of the characterized enzymes from the GH97 family

	Classification	Substrates	Optimal temperature (℃)	Optimal pH	Ion as activator	Hydrolysis linkage	Catalytic mechanism	Catalytic site	Metal ions in the catalytic active center	References
***Qs*GH 97a**	Glucoamylase	pNPαG, Panose, Maltose	50	8	Ba^{2+}, Sr^{2+}	α-1,4, α-1,6	Inverting	Glu 459, Glu 483, and Glu 378	n.c.	This study
**Bt_06 83**	α-Glucosidase	n.c.[a]	n.c.	n.c.	n.c.	n.c.	n.c.	n.c.	n.c.	(Briliūtė et al., 2019)
**PspA G97A**	α-Glucoside hydrolase	pNPαG, Panose, Maltose,Kojib iose	30	7.5	Cl^-	α-1,4, α-1,6, α-1,2	Inverting	Glu 456, Glu 480, Glu 377	Ca, Cl, Mg	(Li et al., 2016a)
**SusB(Bt_37 03)**	Glucoamylase	pNPαG, Panose, Maltose,Kojib iose	45	6.5	n.c.	α-1,4, α-1,6, α-1,2, α-1,3	Inverting	Glu 439, Glu 508, Glu532	Ca	(Gloster et al., 2008; Okuyama et al., 2009)
***Bt*GH 97b(B t_187 1)**	α-Galactosidase	pNPGal,Melib iose	37	6.6	n.c.	n.c.	Retaining	Glu 439, Glu 508, Glu532	Ca	(Gloster et al., 2008; Okuyama et al., 2009)
**Bt_26 20**	α-mannan α-galactosidase	S. pombe α-mannan	n.c.	n.c.	n.c.	α-1,2	Retaining	Asp404, Asp453	n.c.	(Cuskin et al., 2015)
**Bt_36 64**	α-Galactosidase	pNPAra, pNPGal	35	5.5	n.c.	n.c.	Retaining	Asp402, Asp459	n.c.	(Kikuchi et al., 2017)
**Bt_36 61**	Bifunctional β-L-arabinopyranosi dase/α-galactosidase	pNPAra, pNPGal, gum arabic, arabinogalacta n	35	5.5	n.c.	α-1,3-Arabinosyl/α-1,3 and α-1,6 galactosyl residues	Retaining	Asp408, Glu463	Ca	(Kikuchi et al., 2017)
**Bt_32 94**	α-Galactosidase	pNPGal, melibiose, RFOs	60	7	n.c.	α-1,6	Retaining	Asp405, Asp454	n.c.	(Shin et al., 2020)

[a] n.c.: not characterized.

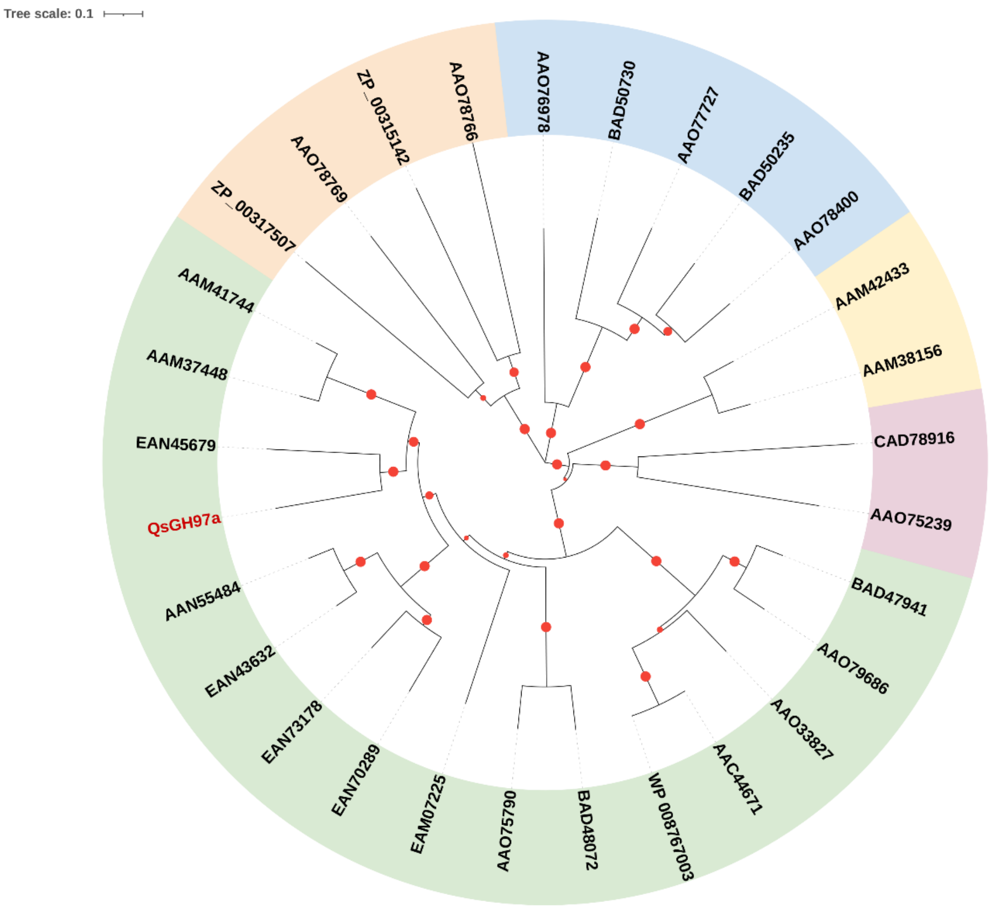

Figure S1. The phylogenetic analysis of GH97. GH97 can be divided into 5 major branches: GH97a, b, c, d, e. GH97a subfamily is presented in the green area, GH97b subfamily in orange, GH97c subfamily in blue, GH97d subfamily in yellow, and GH97e subfamily in purple. The nodes are shown in red.

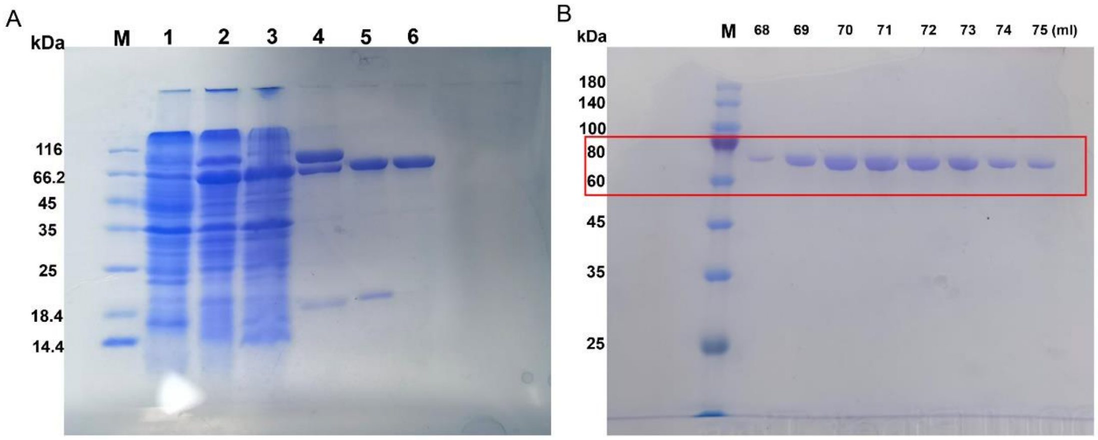

Figure S2. The SDS–PAGE result of QsGH97a purified by Ni-NTA column and Gel filtration chromatography. A: The SDS–PAGE result of QsGH97a purified by Ni-NTA column. M: Marker; lane 1: the supernatant of *E. coli* BL21 plus containing plasmid pSMT3-*QsGH97a* without induction; lane 2: supernatant of the sonication product; lane3: precipitation of the sonication product; lane4: His-sumo-QsGH97a protein; lane5: the recombinant protein after Ulp1 enzyme digestion; lane 6: purified QsGH97a after enzyme digestion. B: The SDS–PAGE result of QsGH97a purified by Gel filtration chromatography. The red box is the part of Figure 3A.

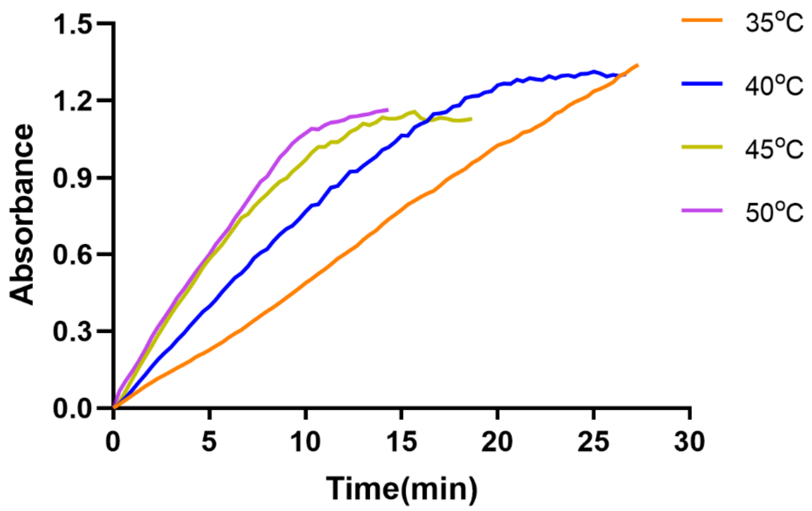

Figure S3. The process of enzymatic reaction under different temperatures from 35°C-50°C. Different colors represent different temperatures.

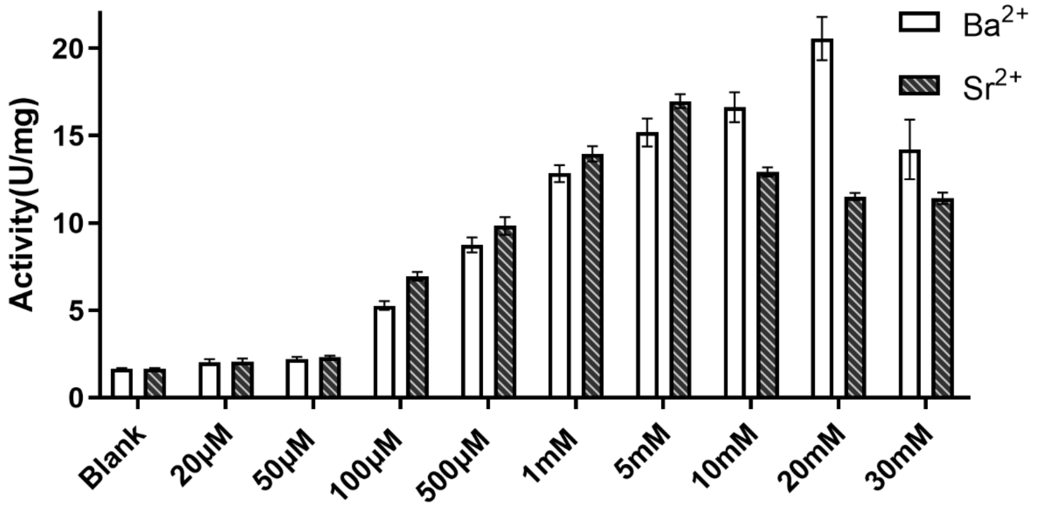

Figure S4. The enzymatic activity in different concentrations of Ba^{2+} and Sr^{2+}. The activity without addition of metal ions was taken as 100%.

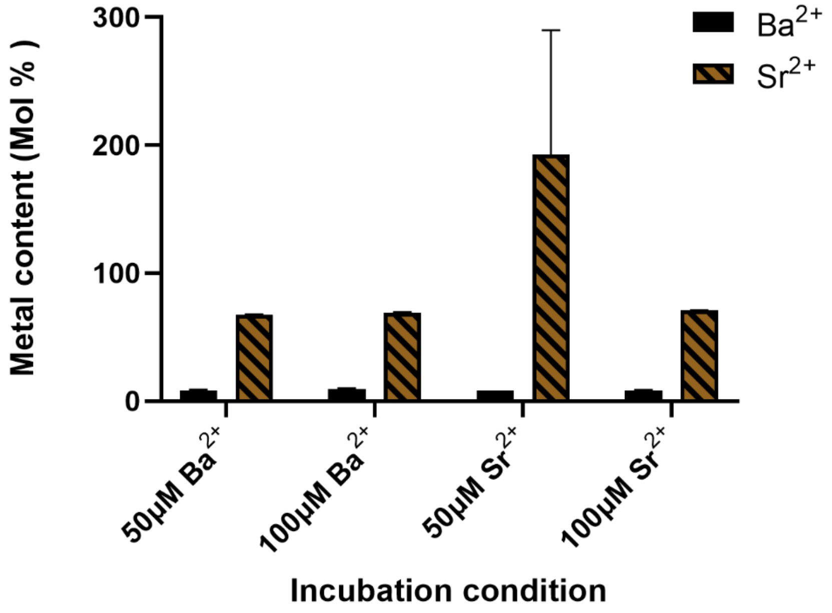

Figure S5. The proportion of metal ions in QsGH97a under different culture conditions. X axis presents the different culture induction conditions of the enzyme, Y axis presents molar ratio of metal ions in per subunit of the enzyme.

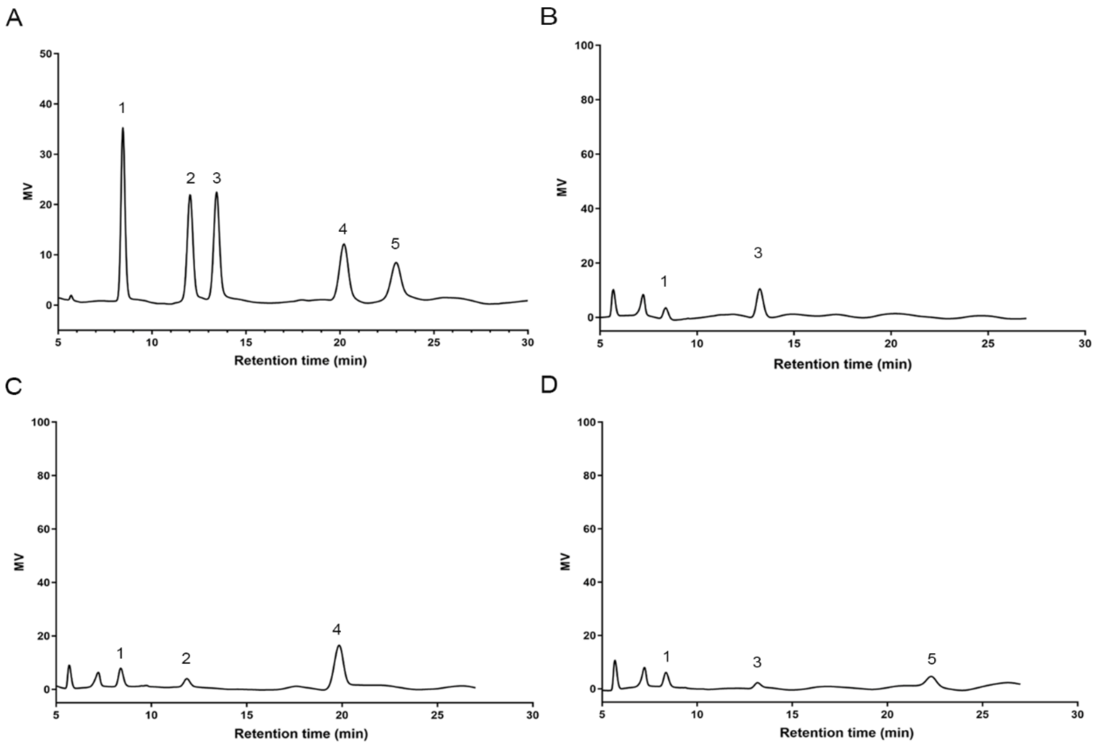

Figure S6. HPLC analysis and enzymatic activity on transglycoside products. (A) Standard curve. Peak 1, glucose; peak 2, maltose; peak 3, isomaltose; peak 4, panose; peak 5, isomaltotriose. (B-D) The result of enzymatic hydrolysis on isomaltose, panose and isomaltotriose, respectively.

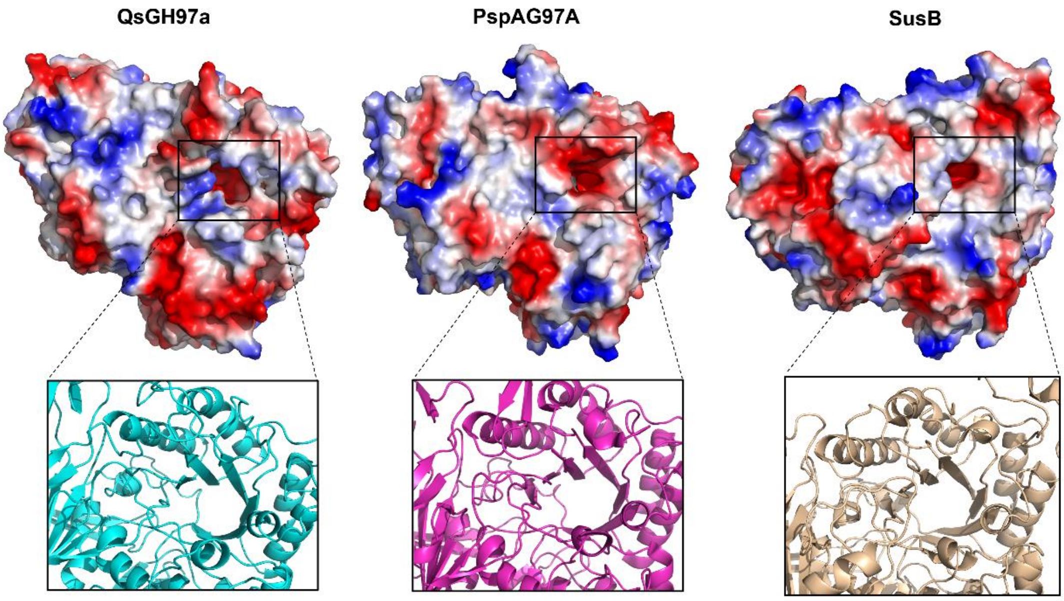

Figure S7. The active site regions of QsGH97a and its homologs. The electrostatic potential surfaces of QsGH97a, PspAG97A (PDB ID: 5HQ4) and SusB (PDB ID: 2JKA)

were shown from left to right.
